# Time-Resolved Mid-Infrared Photothermal Microscopy for Imaging Water-Embedded Axon Bundles

**DOI:** 10.1021/acs.analchem.3c02352

**Published:** 2023-10-25

**Authors:** Panagis D. Samolis, Xuedong Zhu, Michelle Y. Sander

**Affiliations:** † Department of Electrical and Computer Engineering, Boston University, Boston, Massachusetts 02215, United States; ‡ Photonics Center, Boston University, Boston, Massachusetts 02215, United States; § Department of Biomedical Engineering, Boston University, Boston, Massachusetts 02215, United States; ∥ Division of Materials Science and Engineering, Boston University, Brookline, Massachusetts 02446, United States

## Abstract

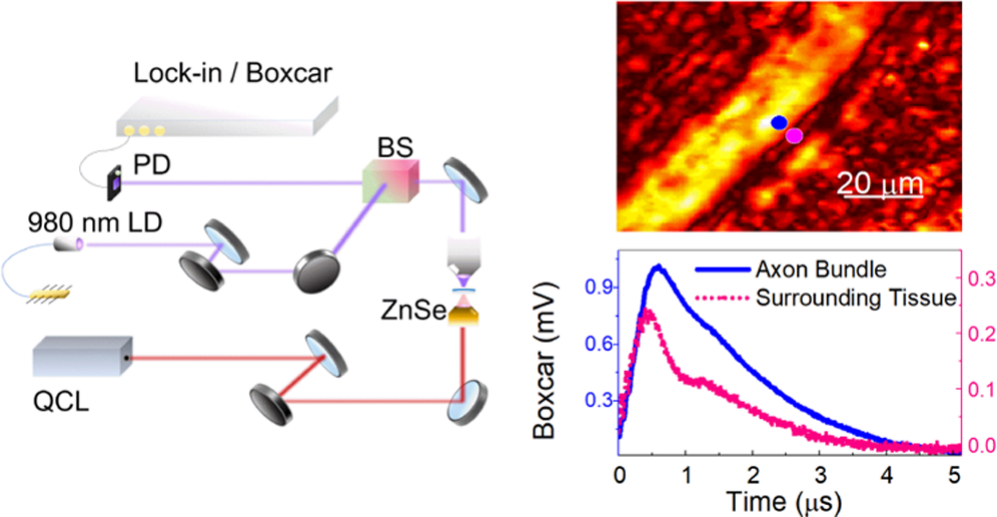

Few experimental tools exist for performing label-free imaging of biological samples in a water-rich environment due to the high infrared absorption of water, overlapping with major protein and lipid bands. A novel imaging modality based on time-resolved mid-infrared photothermal microscopy is introduced and applied to imaging axon bundles in a saline bath environment. Photothermally induced spatial gradients at the axon bundle membrane interfaces with saline and surrounding biological tissue are observed and temporally characterized by a high-speed boxcar detection system. Localized time profiles with an enhanced signal-to-noise, hyper-temporal image stacks, and two-dimensional mapping of the time decay profiles are acquired without the need for complex post image processing. Axon bundles are found to have a larger distribution of time decay profiles compared to the water background, allowing background differentiation based on these transient dynamics. The quantitative analysis of the signal evolution over time allows characterizing the level of thermal confinement at different regions. When axon bundles are surrounded by complex heterogeneous tissue, which contains smaller features, a stronger thermal confinement is observed compared to a water environment, thus shedding light on the heat transfer dynamics across aqueous biological interfaces.

## Introduction

Label-free imaging of chemical signatures with photothermal microscopy^1^ in the mid-infrared (mid-IR) has proven to be a very powerful method for biomedical imaging and has attracted accumulative interest throughout the past years.^2−4^ The ability to target mid-IR vibrational resonances in the molecular fingerprint region with a spatial resolution down to few hundred nanometers^5^ has allowed high-quality imaging of various samples including live cells,^6,7^ pharmaceuticals,^8^ nanostructures,^9^ nanoplastics,^10^ explosives,^11^ as well as material characterization.^12,13^ Pupil engineering for background suppression has enabled the detection of single viruses,^14^ while fluorescence detection of photothermal effects has helped to overcome scattering limitations.^15^ General improvements in mid-IR imaging have been achieved with circular dichroism for examining local tissue changes,^16^ polarization sensitive imaging,^17^ optical interference,^18^ as well as Raman integration.^19^ Further resolution enhancements have been achieved with deep learning.^20,21^ To acknowledge thermal effects, the impact of thermal blurring with modulation frequency^22^ has been investigated. Yet, one ongoing challenge has been the significant water absorption present in the mid-IR.^23^ Mid-IR photothermal imaging of biological samples has been mostly pursued in D_2_O in order to minimize the contribution of the water background. Enhancement of intracellular features and aqueous background suppression was demonstrated with higher harmonic demodulation^24^ and the depth-resolved capabilities from a 3D synthetic aperture in quantitative phase photothermal imaging enabled minimizing the signal from out-of-focus aqueous layers.^25^ In addition, differentiation was obtained based on the larger heat capacity of water leading to smaller phase shift values than for intracellular local signatures.^26^ Recently, imaging speeds beyond video rate for live cell imaging in aqueous H_2_O environments were also demonstrated where spectral differences in the multivariate analysis of hyperspectral images were utilized for water background suppression.^27^

However, in pure H_2_O-embedded complex biological samples with high water content up to 50–90%, the water background absorption can be at similar signal levels to the sample and thus mask a lot of vibrational signatures, e.g., for protein peaks in the Amide I band (where the water absorption coefficient is >1000 cm^–1^).^28^ Thus, while the demonstrated aforementioned methods, including higher harmonic demodulation,^24^ depth sectioning, hyperspectral approaches, and general background subtraction,^25^ can be powerful, they might not be universally applied for all samples, especially with no prior sample knowledge. Since water absorption plays a significant role in the underlying photothermal effects related to electrophysiological processes, it is of significant interest to address the gap of chemical imaging in highly absorbing water environments.

Temporal dynamics can be used as an additional mechanism for differentiation supplementary to absorption, based on the inherent thermal properties of the absorber and its environment. The state-of-the-art method for thermal characterization has been lock-in thermography for thin films.^29^ Selected time-resolved photothermal and photoacoustic methods have been conducted with optical pump–probe measurements in the visible regime.^30,31^ However, mid-IR photothermal microscopy can pave the way for more highly localized characterization of thermal diffusion properties accompanied by chemical information. Up until very recently, photothermal signals have been extracted over an average of many modulation periods, thus losing temporal information. In previous work, vibrational infrared photothermal amplitude and phase signals (VIPPS) imaging has provided contrast from materials with varying thermal properties from membrane interfaces.^32^ The lock-in phase^33^ is a normalized quantity that can provide contrast from changes in heat transfer properties^34^ but cannot characterize the thermal transient responses. Widefield setups have incorporated time studies utilizing virtual lock-in cameras^35^ and high frame rate of CMOS cameras with up to 200 k frames per second along with digital lock-in filters.^36^ In addition, temporal dynamics in quantitative measurements of thermal-induced optical phase shifts have been presented with pump probe delay methods^25,37^ and expanded dynamic range.^38^ However, regarding the study of temporal dynamics, widefield approaches can be limited by the available camera frame rates, which at best provide a time resolution of 5 μs with a frame rate of 200 kHz. Thus, there is a significant benefit in studying transient dynamics at confocal setups where the temporal resolution is determined by the speed of electronics (e.g., oscilloscopes and lock-in amplifiers) with up to hundreds of MHz bandwidths. So far, to the best of our knowledge, there has been only one demonstration of mid-infrared photothermal imaging in a confocal setup that utilized MHz digitization and match filtering to obtain temporal decay curves.^24^ The distinct and faster transient dynamics of different parts of the sample allowed background suppression from D_2_O and identification of weakly absorbing small features like lipid droplets by higher harmonic imaging.^24^ An alternative approach to obtain time-resolved data has been demonstrated with high-speed boxcar measurements, e.g., in Raman spectroscopy with superior sensitivity than lock-in detection.^39^

Here, we present a mid-infrared photothermal microscope integrated with a high-speed boxcar approach to directly measure the photothermal temporal decay characteristics without the need for postprocessing. This enables capturing for the first time expanded time stacks of thermal diffusion processes that fully characterize the evolution of signal in space and time. Further, we will demonstrate time-resolved mid-IR photothermal imaging of biological samples embedded in water, where previous studies focused on heavy water as the background to minimize overlapping absorption bands. With our setup, regular water can be differentiated based on its transient dynamics, whether nearby absorbers are characterized by a faster or slower time decay. Thus, there is no need to manipulate the environment in any way for biological imaging. This can lead to new insights into biophysical phenomena that can be associated with various applications, e.g., infrared neural modulation, where optically induced thermal gradients in water environments contribute to the generation of electrophysiological signaling.^40−42^ In this work, we utilize the enhanced signal-to-noise provided by boxcar detection^39^ in combination with the tunability of a gate window to obtain high-contrast hyper-temporal image stacks of diffusion processes in aqueous interfaces without the need for complex postprocessing. A two-dimensional mapping of the characteristic time decay parameters is presented with high contrast between the extracted axon bundles and the surrounding saline solution based on transient dynamics. In addition, highly localized temporal characterization of heat transfer at the interface allows us to characterize levels of thermal resistance in a quantitative manner and the impact of the axon bundle environment on heat flow. Overall, this methodology paves the way for the novel chemical IR imaging of photothermal effects.

## Experimental VIPPS Imaging Setup and Sample Preparation

**Figure 1:**
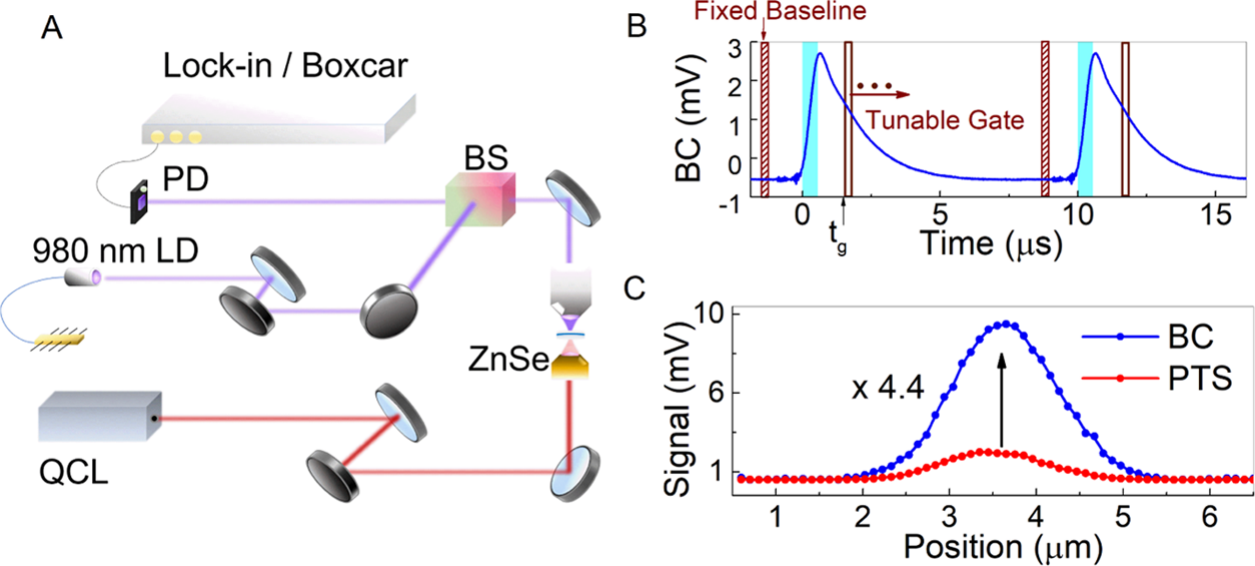
(A) Experimental mid-infrared photothermal setup with boxcar. The QCL pump beam with a repetition rate of 100 kHz illuminated the sample via a ZnSe objective. A 980 nm laser diode (LD) probe illuminated the sample from the top side. The backscattered signal is separated via a 50:50 beam splitter (BS) and focused on a PD which is connected to 600 MHz lock-in with a boxcar tool. (B) Two periods of probe backscatter modulation from a thin water sample. (C) Photothermal imaging signal (PTS) vs boxcar (BC) signal for a 100 nm PMMA bead at *t_g_* = 500 ns features a 4.4-fold SNR enhancement for the BC measurement.

The setup presented in Figure 1A consists of a tunable QCL laser with a wavenumber range of 1580–1740 cm^–1^ emitting a *t*_p_ = 500 ns pulse at a repetition rate of 100 kHz. The pump beam illuminated the sample through a 0.4 numerical aperture (NA) refractive ZnSe objective. An epi-detection setup is implemented for the probe in which a near-infrared continuous-wave laser diode centered at a wavelength of 980 nm is focused on the sample using an NA = 0.65 objective. Power values are set to 1 and 50 mW for pump and probe, respectively, at the focal plane. The focal spot FWHM diameter of pump and probe beam is equal to 6 and 1 μm, respectively. The backscattered signal is collected via a 50/50 beam splitter and focused on a Si photodetector (PD) with 10 dB gain. An extra 5× amplification is applied with a preamplifier and DC filtering with a cutoff frequency of 10 kHz. To filter out the strong DC background and to obtain a flat frequency response for frequencies larger than the fundamental at 100 kHz, the output of the preamplifier is AC coupled to a 600 MHz lock-in amplifier with a 50 Ω input impedance. This provides additional high-pass filtering that prevents voltage overload and compensates for laser noise. The signal is then demodulated with standard VIPPS (Vibrational Infrared Photothermal amplitude and Phase Signals) in order to extract the photothermal amplitude and phase signals. For the preparation of the crayfish axon bundles, crayfish (*Procambarus clarkii*) with 5–7 cm head-to-tail size were purchased from Niles Biological Supplies (Sacramento, CA) and maintained in tap water at room temperature (∼21 °C). The axon bundle (AB) consisting of multiple sensory and motor axons from the first pair of walking legs of the crayfish was dissected and isolated from the remaining tissue in physiological saline containing (in mM): 195 NaCl, 5.4 KCl, 13.5 CaCl2, 2.6 MgCl2, and 10 HEPES (pH 7.4, titrated with NaOH). The extracted axon bundle constitutes various sub-axon bundles, individual axons, as well as elements of connective tissue, e.g., glia cells and collagen fibrils. The individual axons in the bundle have various diameters from ∼5 to ∼ 20 μm. The isolated AB and 2–3 drops of 2 μL of the physiological saline were sandwiched between two one-inch wide CaF_2_ windows.

## Results

### Time Decay Curve Acquisition via Boxcar Measurements

To capture temporal dynamics associated with photothermal heating and diffusion, a boxcar (BC) configuration is installed for extracting the time decay profile and averaged over 2040 periods with a time resolution of 10 ns. A representative boxcar measurement for a 100 nm PMMA bead in air is shown in Figure 1B. The regime of heating is highlighted in the figure with cyan background, mapped to the time windows of signal increase while diffusion processes are associated with the decay. By tuning the gate start time *t_g_* of the boxcar gate window and fixing the boxcar baseline window at time intervals with no signal (see Figure 1B), the boxcar outputs for selected points in time are obtained. The latter can be used to acquire hyper-temporal image stacks by raster scanning the sample stage in two dimensions (2D) at a fixed gate time *t*_g_ with a pixel dwell time of 28 ms. The time step between each image is fixed by the duration of the boxcar gate window. Considering the relatively slower heat decay with respect to the excitation, a gate time window duration equal to half the pump pulse duration (250 ns) is found to be sufficient to cover the entire frequency range of the obtained transient curves.

For this study, the absolute value of the boxcar output was used to reconstruct the digital 2D boxcar image. The time window of signal decay is termed the diffusion window for the rest of this paper. In addition, the tuning of the boxcar gate window allows the selection of the peak signal contributions, which can enhance the overall measurement sensitivity, as demonstrated in Figure 1C. A clear 4.4-fold improvement in the signal-to-noise ratio (SNR) of the linescan across a 100 nm PMMA bead is reported, from 126 to 549. A similar almost 4.3-fold SNR improvement was demonstrated by harmonic match filtering^24^ where the SNR scales with the number of collected harmonics. However, the number of detectable harmonics can be sample-dependent and is limited by the detection threshold. The SNR in boxcar acquisition scales with √*N*, where *N* corresponds to the number of boxcar periods that can scale theoretically to very large values with no limitation. To the best of our knowledge, boxcar acquisition has shown a very high SNR for 100 nm PMMA particles of 549 without the need for postprocessing and external filtering compared to an SNR of 230 for 300 nm PMMA particles.

### Imaging of an Axon Bundle–Water Interface

**Figure 2:**
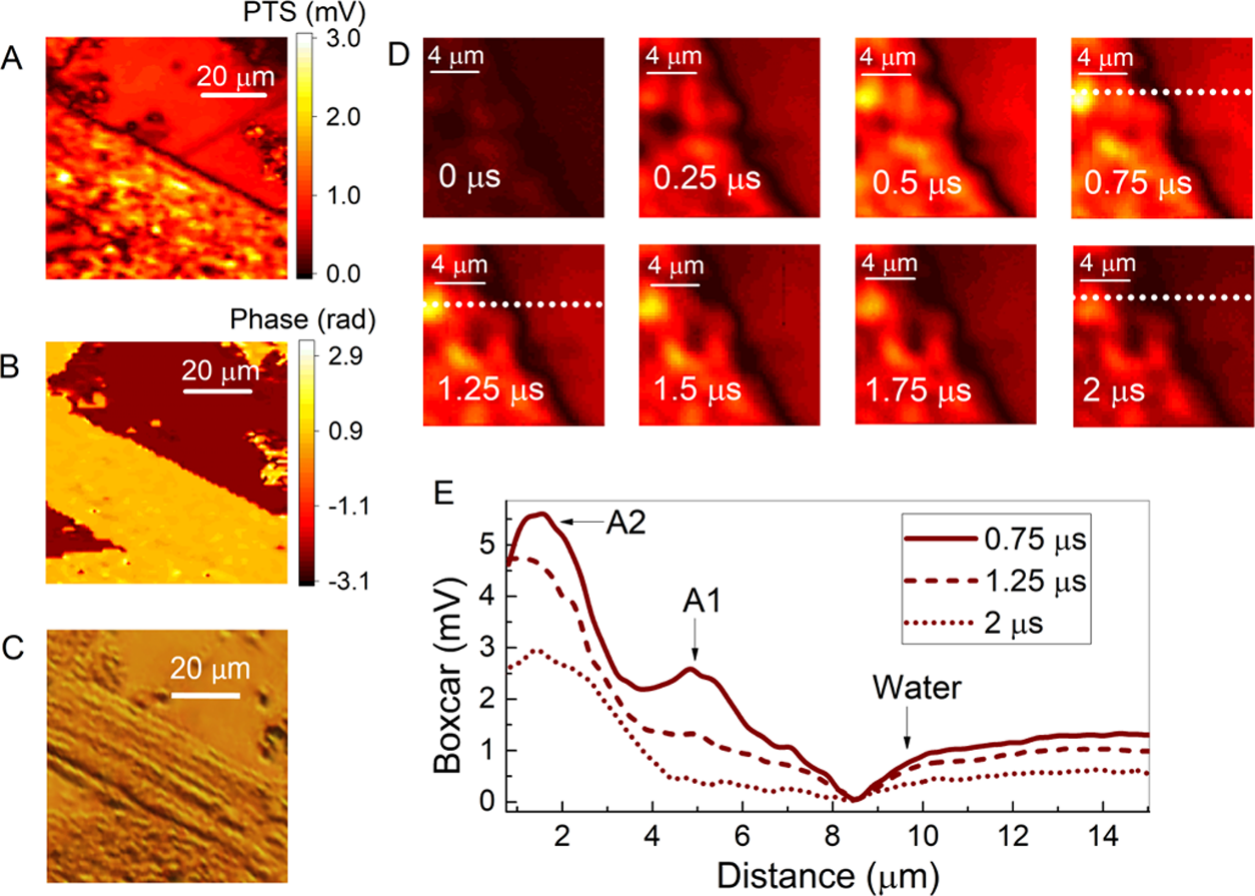
Photothermal (A) PTS, (B) phase, and (C) optical microscope images of an extracted axon bundle in a saline environment. (D) Time stack of the boundary interface from *t*_g_ = 0 μs to *t*_g_ = 2 μs separated by 250 ns. (E) Evolution of the horizontal cross section at *y* = 10 μm for time points *t*_g_ = 0.75 μs (solid brown), *t*_g_ = 1.25 μs (dashed brown), and *t*_g_ = 2 μs (dotted brown) with two highlighted points at the axon bundle (A1, A2) and water.

VIPPS measurements of amplitude (PTS) and phase imaging, cross-registered with an optical microscope image, were performed on an isolated sub-axon bundle (AB) surrounded by saline solution, see Figure 2A–C. High absorption from both the AB and the saline environment is reported in the PTS image with the water signal being 2–2.4 times smaller than the average AB signal. The phase image in Figure 2B features an enhanced contrast at the interface. The boxcar measurements are performed on a 15 by 15 μm area where the boundary between the AB and saline is clearly visible. Images at varying points in time were obtained by tuning the boxcar gate start time from *t*_g_ = 0–2 μs, see Figure 2D. An increase of photothermal signal due to heating occurs mainly before *t*_g_ = 0.5 μs until the photothermal signal starts to decrease due to dominant diffusion processes after *t*_g_ = 0.5 μs. The BC temporal evolution from *t*_g_ = 0.75 to 2 μs of the cross section across the AB boundary located at *y* = 10 μm (see the dashed white line in BC images) is shown in Figure 2E.

A clear broadening of the interface boundary is visible (darker shaded black region in Figure 2D) within a time interval of 1.25 μs during the diffusion process. At the cross section located at *y* = 10 μm (see white dashed line), three points of interest are selected, each corresponding to an area with a notable change in spatial gradient. Two of those points A1 and A2 are in the AB interior (positioned by 3.2 and 6.4 μm from the interface, respectively). The third point denoted as Water is distanced 1.2 μm from the interface. A 1.6-fold broadening of the peak width at A2 in the boxcar signal (the FWHM increases from 1.7 to 2.8 μm from 0.75 to 2 μs in Figure 2E) indicates thermal blurring effects and heat transfer toward the nearby AB environments. A similar change in the AB morphology is observed closer to the interface, where the signal associated with A1 undergoes a strong decay. This effect in combination with interface broadening indicates heat transfer from the AB toward the water and nearby environments.

**Figure 3:**
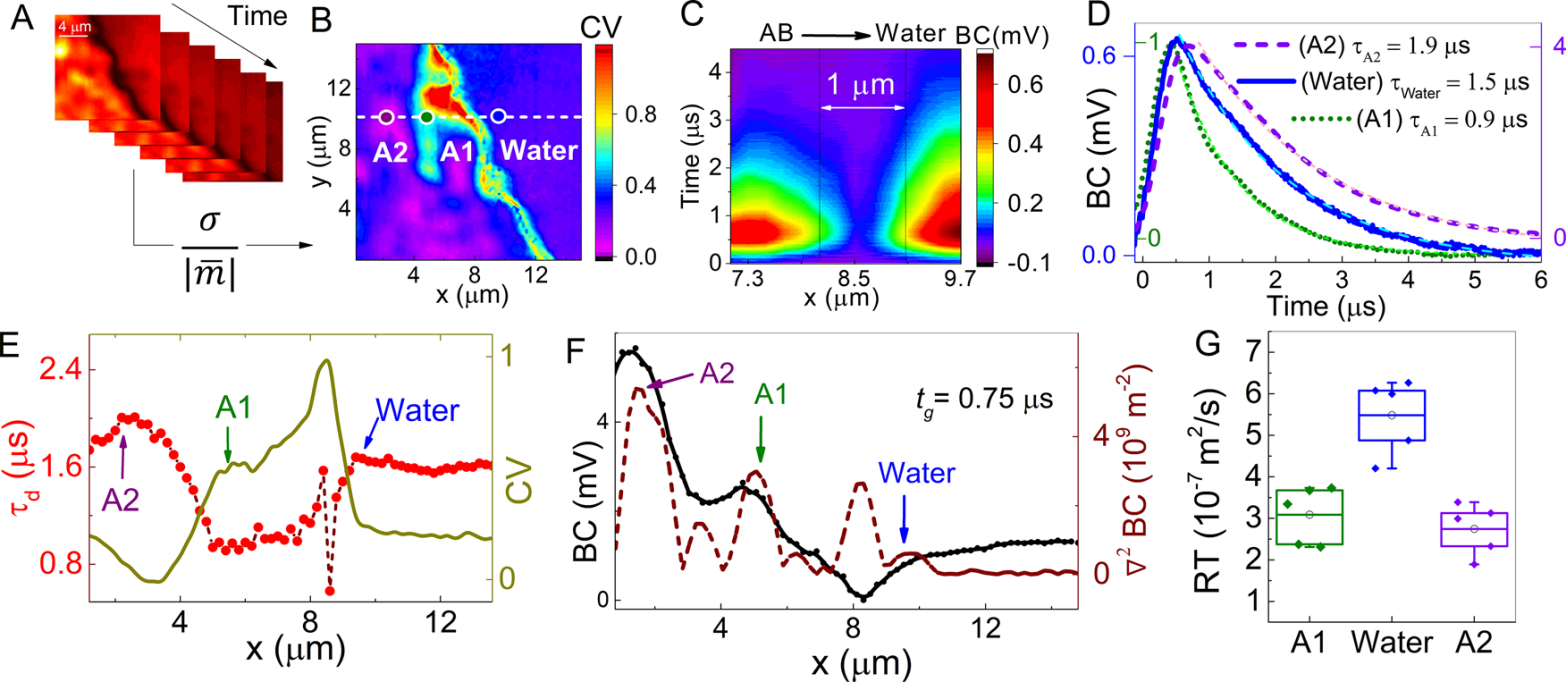
(A) Time stack of the AB/water boundary, corresponding to Figure 2D. (B) Coefficient of variance (CV) image of AB/water boundary highlighting areas with fast time decay rates. Dots indicate selected points of interest for water, the AB near the interface (A1), and the AB in the interior (A2). (C) Evolution of time curves across the interface. (D) Time curves with time decay parameters (*τ_d_*) for water (blue) and AB, A1 (green), and A2 (violet) with *τ*_A2_ > *τ_Water_* > *τ_A1_*. (E) Spatial evolution of τ_d_ across the interface (red circle) along with CV spatial profile (solid dark yellow). (F) Spatial evolution of the BC signal (solid black) and its Laplacian (dashed brown) across the interface at *t*_g_ = 0.75 μs. (G) Rates of transfer at selected points: water (blue), A1 (green), and A2 (violet) with *RT_water_* > *RT_A2_*, *RT_A1_*.

To spatially visualize the temporal dynamics, the areas characterized by a varying time decay parameter can be enhanced by introducing a coefficient of variance (CV) at the measured image time stack, see Figure 3A. This is defined as the ratio of standard deviation σ over the absolute value of the mean |*m*| per pixel as . The CV image is shown in Figure 3B. The average value of the CV signal at the water background is ∼0.3 and is relatively constant. Significant peaks are observed at the interface with CV > 0.3 and for parts of the AB interior (see yellow), where previously low photothermal confinement was reported. In addition, regions with CV < 0.3 are found in deeper parts of the AB interior (see blue regions). The previously selected points of interest Water, A1, and A2 correspond to CV values CV ∼ 0.3, CV > 0.3, and CV < 0.3, respectively. A series of time curves collected across the boundary with a step size of 200 nm are shown in Figure 3C. The latter figure demonstrates a decrease of the time decay parameter *τ_d_* from 1.5 to 1 μs over a 1 μm area near the interface. The individual time curves of the points of interest for Water, A1, and A2 are shown in Figure 3D. The time decay parameter during the diffusion window is quantified by the 1/e margin from the peak to minimum signal and was found to be equal to *τ_d-A1_* = 0.9 μs, *τ_d-A2_* = 1.9 μs and *τ_d-Water_* = 1.5 μs. Thus, the axon bundle region in close proximity to A1 is characterized overall by a faster decay and is significantly enhanced in the CV image. This method offers good contrast for a region that in standard photothermal imaging is not easily differentiated from its nearby AB environments. In addition, the CV values can be interpreted as a mirrored evolution of the time decay *τ_d_*, cf. Figure 3E. As the time decay and thus the absolute value of the CV can vary based on water thickness and sample preparation, the focus is here on the relative CV difference and how that leads to the differentiation of features in the same environment. This provides a two-dimensional visualization of both fast (higher CV) as well as slow (lower CV) decaying regions and enables mapping τ_d_ without the need for a pixel-by-pixel exponential fitting, which could require a significantly larger time stack.

### Water Differentiation and Rate of Transfer

In general, considering that water absorption coefficients *μ_abs_* are larger than >1000 cm^–1^ at vibrational bands of lipids and proteins (e.g., around 3 and 6 μm, respectively), it can be challenging to control the level of water background especially in complex samples like our 5 cm long axon bundles. Thus, background subtraction cannot be applied universally across samples using photothermal amplitude images when the water signal levels are also present in the interior of the AB, as can be seen by all presented boxcar and photothermal images. Here, water is found to have a distinct and consistent transient response with a time decay parameter around *τ_d-water_* = 1.5 μs. This can be mapped to a distinct CV signal; specifically, 75% of CV_water_ values fall between 0.22 and 0.35. The equivalent CV_AB_ values fall in a more dispersed range from 0.07 to 0.57 (up to 3.8 times larger). When comparing the equivalent range for the corresponding PTS values, 75% of PTS_water_ falls between 0 and 0.74 mV, while the PTS_AB_ values are found to be again more dispersed but only by a factor of 1.9, from 0 to 1.4 mV.

Previously, it was reported for biological samples, specifically brain cancer cells in a D_2_O bath, that the water background could be effectively distinguished by different thermal decay rates extracted through MHz digitization from photothermal measurements,^24^ which is consistent with our finding. In the aforementioned study, the background differentiation relied on higher harmonic demodulation due to the fact that the absorbing features of interest had faster transient dynamics than the surrounding heavy water, which led to a relatively stronger higher harmonic component of the signal. However, we found that this is not necessarily applicable for all samples, especially complex structures like the imaged axon bundle, where features of interest (A1 and A2) had decay rates faster and slower than water. Thus, in this work, a methodology of strong water background suppression is presented that does not rely on the condition that biological features have a faster decay rate than the background. Here, instead of plotting the thermal decay rates associated with each position in the sample directly, which can be computationally more complex, the CV image is evaluated. The contrast provided by the presented CV images based on transient dynamics enables identifying areas with faster and slower decay times. Additionally, in the CV images, water is represented by a more homogeneous background and a higher interface contrast compared to a conventional photothermal amplitude image, allowing for an effective separation of the water background. In the Supporting Information (see Figure S1), the PTS images and CV images after having subtracted the median PTS and CV value of the water region, respectively, are shown. In the resulting CV image, the water is characterized by a dark region that is readily identifiable compared with the water region in the PTS image. At the same time, the interface is enhanced in the CV image. Thus, the water background is found to be more distinguishable in the CV image. Considering that biological tissue features a strong water content (up to 75%), the contrast provided by the CV image is not based on inherent distinct spectroscopic signatures but rather differentiates features with significant overlap of chemical content based on different transient dynamics and based on their material properties and thermal resistance. Overall, this provides a novel method for differentiating sample features with similar absorption to that of water, especially for investigating regions near interfaces.

To further investigate the thermal dynamics, in analogy to the thermal diffusion equation, we define the rate of photothermal signal decay as , where *RT* corresponds to the rate of transfer in m^2^/s and BC to the boxcar signal. Similarly, to the thermal diffusivity, *RT* quantifies the rate of heat transfer speed toward thermal equilibrium at a specific location. In Figure 3F, the BC is plotted with the two-dimensional Laplacian at *t*_g_ = 0.75 μs, where the points of interest (A1, A2, and Water) coincide closely with the peak values of ∇^2^(BC), as denoted by arrows. By combining spatial and temporal information, the normalized rate of transfer for each area is calculated for five time points during the diffusion period (between *t_g_* = 0.75–2 μs); see box chart in Figure 3G. The box boundaries in the *y* direction represent the 25–75% statistical margin and the open circle together with the middle line indicates the mean value for each set. *RT_A1_* and *RT_A2_* feature comparable mean values at 3 × 10^–7^ and 2.7 × 10^–7^ m^2^/s, respectively, while water has a higher rate of *RT_water_* = 5.4 × 10^–7^ m^2^/s. Points A2 and A1 mark absorbing features that exchange heat with their environment by diffusion, leading to significant levels of blurring. This was already observable by the increasing FWHM in the boxcar signal in Figure 2E, indicating that heat is diffusing away from both A1 and A2 toward the surrounding environment of the AB.

To interpret the higher *RT* value for water, numerical simulations were performed (see Supporting Information Figure S2) on an equivalent interface. We found that depending on the feature size, the transient response can either decay faster or slower than the surrounding water. The numerically estimated *RT* values of those hotspots were found to be consistently between 2.3 and 3.5 × 10^–7^ m^2^/s. The numerical *RT* in close proximity to the interface at the lower temperature side (water, 1 μm away) was found to be larger by a factor of 1.5 around 6.6 × 10^–7^ m^2^/s, which agrees well with the 1.8-fold higher experimental *RT* value measured for water compared to *RT* values at points A1 and A2. Since the lower-temperature region of the interface in both experiments and simulations is shown to have a larger *RT* and higher speed toward thermal equilibrium, it can be concluded that heat diffusion from the axon bundle hotspots toward the water is not sufficiently slowing down the heat decay at the water side, allowing it to reach thermal equilibrium at a faster rate. This is also visualized in Figure 2E, where A1 has higher signal than water at *t*_*g*_ = 0.75 μs, but a lower one than water at *t*_*g*_ = 2 μs, indicating that heat from A1 is not entirely diffusing toward the water side but also to the nearby AB areas as well.

### Interior Axon Bundle Imaging

**Figure 4:**
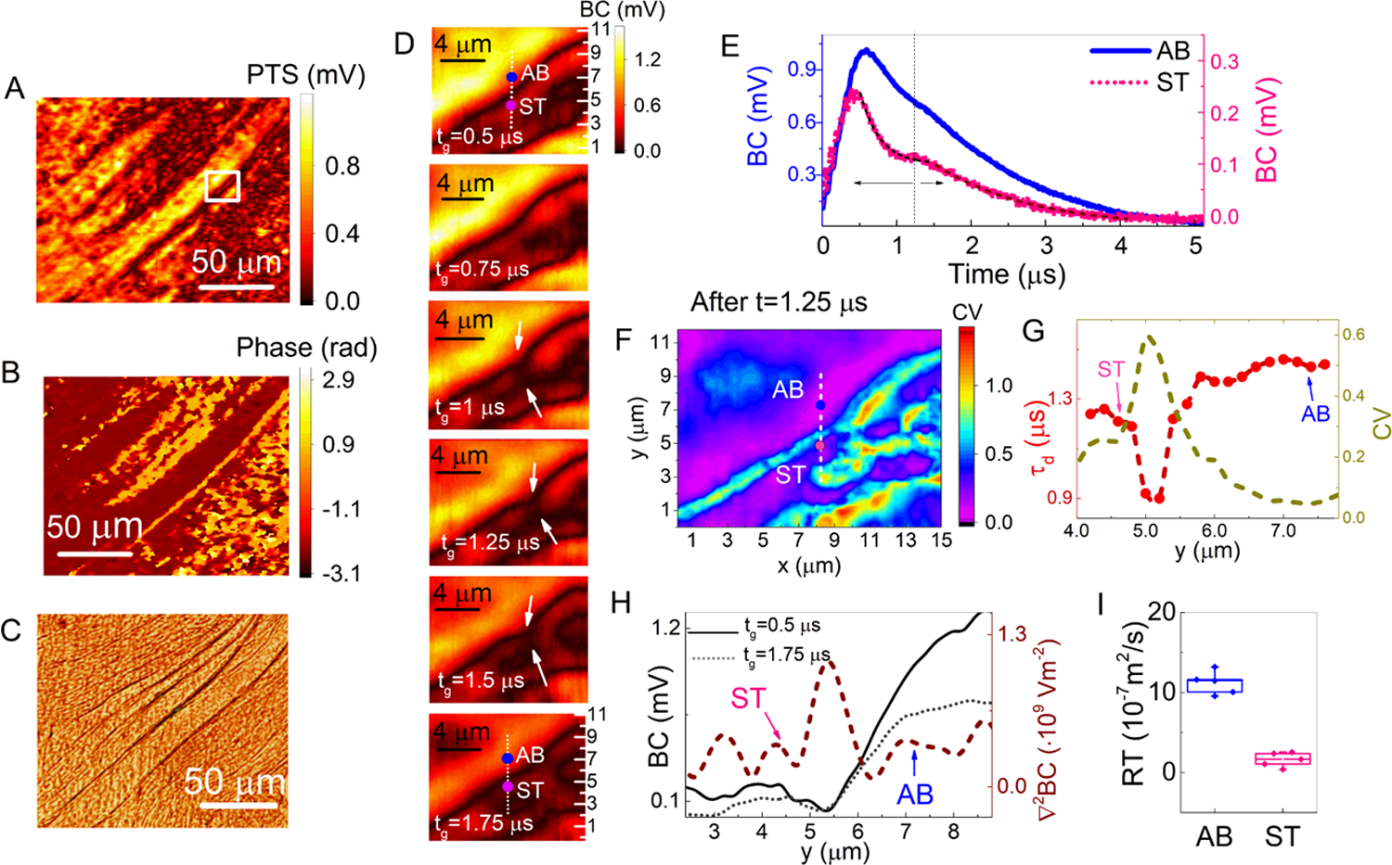
(A) PTS, (B) phase, and (C) optical microscope image of a crayfish sub-axon bundle. (D) Time stack of the boundary interface from *t*_g_ = 0.5 μs to *t*_g_ = 1.75 μs. The blue dot highlights point (AB) in sub-axon bundle and the pink dot highlights point (ST) in the surrounding tissue. Arrows highlight dip formation in the surrounding tissue (ST). (E) Time curves at AB (solid blue) and ST (dotted pink). (F) CV image of the AB boundary with enhanced features in the ST region (see pink dot). (G) Spatial evolution of τ_d_ (red circle) across the interface along with the CV profile (dashed dark yellow) at the white dashed line, highlighted in (F). (H) BC signal across the interface at *t*_g_ = 0.5 μs (solid black) and *t*_g_ = 1.75 μs (dotted black) alongside the Laplacian at *t*_g_ = 0.5 μs (dashed brown). (I) Rates of transfer for ST (pink) and AB (blue) with *RT_AB_* > *RT_ST_*.

Next, a different case of a sub-axon bundle sample was studied that was still embedded and not isolated from its surrounding tissue. The PTS and phase images acquired with VIPPS along with the cross-registered optical microscope image of a sub-axon bundle are shown in Figure 4A–C. The PTS image demonstrates regions of high signal corresponding to sub-axon bundle features, embedded by areas with a more inhomogeneous signal distribution and ca. 3–5 times lower levels compared to the sub-axon bundles. This surrounding environment will be referred to as the surrounding tissue (ST) for the rest of the paper. The phase signal highlights the boundary of the AB structure with a high contrast. BC images of the AB interface highlighted with a white box in Figure 4A are shown for *t*_g_ = 0.5–1.75 μs (see Figure 4D). Time curves were collected at two points from each side of the interface, namely, point AB (Axon bundle side, see blue dot in Figure 4E) and ST (surrounding tissue side, see pink dot in Figure 4E) distanced by 1.8 and 1 μm from the edge, respectively. At the tissue side, a double exponential decay rate is observed with a fast decay of *τ_d_* = 200 ns (for 0.5 μs ≤ *t*_g_ ≤ 1.25 μs) followed by a slower decay of *τ_d_* = 1.2 μs. The time point *t*_g_ = 1 μs simultaneously maps the formation of a dip in the BC images in the surrounding tissue region observed from *t*_g_ = 1–1.5 μs (see arrows in Figure 4D). This effect is not observed on the side of AB, which shows a more consistent time curve shape for locations distanced up to 2 μm from the edge. The detected delay in the ST time curve indicates the effects of thermal resistance. By plotting the CV image for points in time after *t*_g_ = 1.25 μs (see Figure 4F), areas with fast and slow decay parameters are highlighted. On the ST side, darker (purple-shaded) oval-shaped features appear with an almost ∼1 μm diameter that are not visible in the standard photothermal images. The overlap of the fitted decay rates *τ_d_* and CV values is shown in Figure 4G, where the correlation between high CV and low τ_d_ (and vice versa) is confirmed. The BC signals across the interface at *t*_g_ = 0.5 μs and *t*_g_ = 1.75 μs are shown in Figure 4H alongside the Laplacian at *t*_g_ = 0.5 μs, with distinct Laplacian peaks corresponding to the points AB and ST seen neighboring the interface. During this 1.25 μs time window of diffusion, the boxcar signals at ST and AB decreased by a factor of 2 and 1.5, respectively. Further, a significantly larger difference between the two interface environments is also reported in the rate of transfer values for which the mean *RT_ST_* ∼ 0.8 × 10^–7^ m^2^/s is lower than *RT_AB_* ∼ 1.1 × 10^–6^ m^2^/s by a factor of 13.7, as seen in the box chart figure in Figure 4I.

The AB area features consistently a higher BC signal with respect to the ST area throughout the time window of interest. As a result, heat is expected to continuously diffuse away from the AB.

In order to better interpret the behavior at the ST region, numerical simulations were performed (see Supporting Information Figure S2) of an interface between a homogeneous environment, analogous to the AB area, that is in contact with spherical features (representative for the features highlighted in the ST region). The presence of spherical features at the interface numerically translated itself into a double-exponential decay at the lower-temperature interface side. Multi-decay time curves similar to the one characterizing the surrounding tissue have also been previously observed in small features like lipid droplets^24^ where an initial fast decay was followed by a slower one. In previous photothermal work, the time decay has been characterized by several groups based on Fourier’s law of heat conduction from which the characteristic thermal decay times constant is proportional to (*C*_V_*·V*)/(*h·A*),^24,43^ where *C*_V_ corresponds to the volumetric heat capacity of the absorber, *V* is its volume, *h* is the heat transfer coefficient to the local environment, and *A* is the surface area. Based on our numerical findings, smaller feature sizes result in an overall smaller volume-to-surface area ratio and consecutively to a faster time decay. As a result, the small dimension of the feature can be responsible for the initial fast decay, while the following slower decay can be attributed to the delayed heat transfer from the surrounding warmer environment (in our case the axon bundle). Thus, the delay of heat transfer is more pronounced for smaller features, with higher curvature due to the small thermal contact surface area, reducing the local *RT* value. Overall, both experimental and numerical results showed that depending on the composition of the tissues and cells close to an interface, the speed toward reaching thermal equilibrium can vary by up to an order of magnitude due to a higher effective interfacial thermal resistance.

Aside from inherent thermal resistance at the membrane interface, surface contact and molecular interaction and geometry could also be considered. Even though the detailed physical mechanisms can be complex, heat transport at water interfaces can be enhanced via the presence of hydrogen bonds.^44,45^ In addition, adsorption of ordered water molecules around nanoparticles can enhance the hydrophilicity,^45^ which increases the thermal conductance^46^ due to strong nanoparticle–fluid interactions.^47^ Such interfacial layers can have up to 50%^48^ or even 1 order of magnitude higher thermal conductivity compared to bulk values.^49^ However, higher curvature of particles can inhibit hydration,^50,51^ which can result in a decrease of the thermal conductance.^46^ This agrees with our results that the smaller surface area of tissue features in direct contact with the AB, including smaller sub-axon bundles, single axons, glia cells, collagen fibrils, and other elements of connective tissue, can result in a weaker interfacial water interaction strength and an enhanced thermal resistance compared to the elongated and larger sized, more isolated axon bundle. Overall, the environmental geometry as well as the level of hydration and water contact thus can influence the underlying heat transfer dynamics, underlying the importance of studying biological samples in their natural physiological saline-water-based environment compared to heavy water or others. It should also be noted that the chemical composition of biological interfaces is of course more complex with the boundaries (e.g., lipids) featuring different properties than the interior bulk, which will impact the thermal resistance since the interaction of proteins or lipids with neighboring environments can vary significantly. Whether any of the observed effects are natural to the sample or photothermally induced externally by the mid-IR pump remains to be explored further.

## Conclusions

Contrast in VIPPS imaging not only relies on the relative photothermal amplitude signal difference but can be enhanced by interfacial temperature gradients that can be captured by lock-in phase imaging. Although the absorption of water and respective proteins of biological tissues as well as the bulk material thermal diffusivities and thermo-optic coefficients can be similar, the thermal gradients at such an interface are unique due to the presence of interfacial resistance effects. The presented time-resolved photothermal imaging and boxcar measurements offered a visualization of the temporal evolution of the water interface gradients during heating and cooling, shedding light on the underlying thermal dynamics with enhanced signal-to-noise. Hyper-temporal image stacks of diffusion processes across the interface of extracted sub-axon bundles embedded in physiological saline were collected for the first time. This technique enabled a two-dimensional mapping of the heat decay profile without complex postprocessing and thus provided an effective method for differentiating features with similar signal levels from the water background. The coefficient of variance of hyper-temporal image stacks highlighted the fast and slowly decaying features, whose signatures and morphology can otherwise be buried in conventional photothermal imaging. Further, this provided an effective means to differentiate the saline water bath from the sample features of interest. In addition to transient recordings, empirical methods such as the parameter of rate of transfer utilize spatial information to provide a complete picture of the thermal diffusion processes. It was demonstrated that the heat transfer in an axon bundle surrounded by water was significantly different compared with axon bundles surrounded by tissue features, with the latter demonstrating higher levels of interfacial thermal resistance. The interplay of heat transfer between different areas of the AB interfaces was studied in detail. Overall, photothermal imaging with boxcar gating enabled high-contrast mid-IR imaging in water and provided the ability to differentiate features with similar absorption and chemical content based on different transient dynamics. This research paves the way for a deeper understanding of the role of photothermal effects in neurons and cells. Further, capturing the interfacial temporal dynamics coupled to the inherent thermal properties can enable deeper insights into fundamental heat transport processes in biological and other samples as well as provide a label-free module for imaging cells in their physiological environment.

## Supporting Information Available

Statistical analysis of CV versus PTS images for water separation and numerical simulations of aqueous interfaces for varying levels of temperature gradients and varying levels of interfacial feature sizes (PDF)

## Abbreviations

VIPPS: vibrational infrared photothermal and phase signals imaging
PTS: photothermal signal
CV: coefficient of variance
BC: boxcar
AB: axon bundle
ST: surrounding tissue
RT: rate of transfer
QCL: quantum cascade laser
PD: photodetector
