# Supplementary material for: Time-Resolved Mid-Infrared Photothermal Microscopy for Imaging Water-Embedded Axon Bundles
Source: Anal Chem. 2023 Oct 25;95(45):16514–21. doi: 10.1021/acs.analchem.3c02352 (PMC10652238; doi:10.1021/acs.analchem.3c02352)
Supplement: Supplementary file 1 — ac3c02352_si_001.pdf [file ac3c02352_si_001.pdf]

Supporting Information

Time-resolved mid-infrared photothermal microscopy for imaging water embedded axon-bundles

Panagis D. Samolis[1,2], Xuedong Zhu[2,3], and Michelle Y. Sander[1,2,3,4,*]

[1]Department of Electrical and Computer Engineering, Boston University, Boston, MA 02215, USA

[2]Photonics Center, Boston University, Boston, MA 02215, USA

[3]Department of Biomedical Engineering, Boston University, Boston MA 02215 USA

[4]Division of Materials Science and Engineering, Boston University, Brookline, MA 02446, USA

*Corresponding author: msander@bu.edu

Table of Contents

Section SI: Subtraction of water background separation …S2

Section SII. Numerical simulations of heat transfer across aqueous interfaces …S3

Section SIII. References …S6

SI. Subtraction of water background

A statistical analysis of the coefficient of variance (CV) and photothermal amplitude (PTS) images presented in Figure 3 of the main text of the axon bundle (AB) and water interface demonstrated a higher level of homogeneity of the water background for the CV image compared to the photothermal amplitude image. Specifically, in the interfacial region, 75% of the PTS_{AB} values fall in a range from 0-1.4 mV, whereas 75% of the PTS_{Water} values cover a 1.9 times smaller range from 0-0.74 mV. At the same time, 75% of the CV_{AB} values are between 0.07-0.57, whereas 75% of CV_{Water} values fall into a 3.8-fold smaller range from 0.22-0.35. This smaller value range of the CV values at the water region allows for differentiation in identifying the water background and separating it from the axon bundle. In Figure S1A and Figure S1B the median CV and PTS values of the water region were subtracted from the CV and PTS images and the absolute value of the difference images is presented. The linescans (y=10 μm) across the interface of each image are shown in Figure S1C. Here, the interface boundary around 8 – 10 μm is enhanced in the CV image. Further away from the AB (y >11 μm), a constant close to zero value characterizes the saline bath region, thus, showing an improved differentiation and suppression of the water background than in the PTS image.

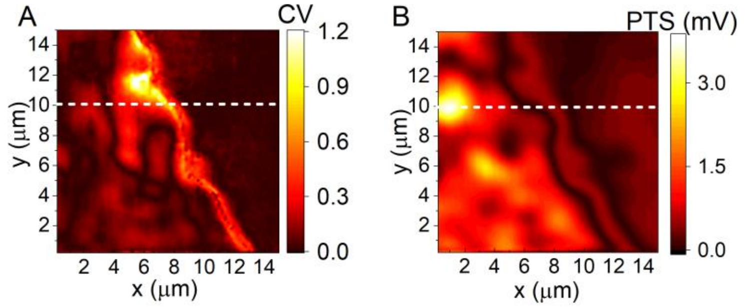

Figure S1. (A) Coefficient of variance image of the axon bundle and water interface after subtraction of the median CV value at the water region. (B) Photothermal amplitude image of axon bundle and water interface after subtraction of the median PTS value at the water region. (C) Linescans across the interface at y=10 μm for CV (black circle) and PTS image (blue triangle) after water background subtraction, where the CV data shows a strong differentiation between the AB and the suppressed water region (y >11 μm).

SII. Numerical simulations of heat transfer across aqueous interfaces

Numerical simulations of heat transfer across different interfaces were conducted with the MC Matlab open source simulation tool for heat diffusion[1]. A 40 x 40 x 40 μm cuboid was used for the simulation consisting of a μm thin aqueous absorbing layer sandwiched between two CaF_2 windows, as seen in Figure S2. The $1/e^2$ radius of the absorbing beam is set at 5 μm. The beam illuminates the center of the layer with a pulse duration of 500 ns and a peak power of 20 mW. The layer thickness is estimated based on the experimentally obtained time decay parameter for water which in the proximity of the axon-bundle was found to be on average close to 1.5 μs. Thus, a thickness of 1.2 μm is chosen to match the experimentally observed values accordingly.

The geometries in Figure S2A and Figure S2B are representative of the interface images presented in Figure 3 and 4 of the main text, corresponding to the axon bundle (AB)-Water and axon bundle - Surrounding tissue interface respectively.

The experimentally extracted PTS signal is proportional to the dn/dT of the thermo-optic coefficient of the absorber. Given that in the experimental data presented in Figure 2 and 3 of the main text, the AB photothermal signal is around two times larger than water, the actual temperature ratio between AB and water is estimated to be between 2 and 4 times larger (since the thermo-optic coefficients of protein[2] and water are fairly similar). Thus, for Figure S2A, an interface was modeled by defining two regions in thermal contact with 3:1 ratio of absorption coefficients μ, with μ_{water} = 2300 cm^{-1}, equal to the coefficient value of liquid water at the Amide I band[3], and $\mu_{AB}=3\cdot\mu_{water}$= 6900 cm^{-1} (see the interface between the water region in blue and the AB in purple in Figure S2A). Since the AB is expected to have strong water content (up to 75%[4]), the thermal properties, including the thermal conductivity κ and volumetric heat capacity C_v, were set equal to those of bulk water, as seen in the parameter Table 1 in Figure S2. However, experimentally there is a variety of transient thermal dynamic responses in the interior of the AB accompanied by an inhomogeneous distribution of the photothermal signal with hotspots like A1 and A2 (see Figures 2 and 3 of the main text). In the simulations, we define two spherical features with two different radii, ABF_1 with r=400 nm and ABF_2 with r=1200 nm, whose thermal properties more closely resemble the values attributed to proteins[5-8] and lipids[9-13] including $\kappa = 0.2\ W\cdot m^{-1}\cdot K^{-1}$, and C_v=2.4 $MJ\cdot m^{-3}\cdot K^{-1}$. These points are defined in analogy to points like A1 and A2 from Figure 3 of the main text. The point of interest at the water region is referred to as Water and distanced 1 μm from the interface, similar to the experimental studies. Experimentally, points like A2 can have a signal up to 4 times larger than water, thus μ_{ABF} is set to $4\cdot\ \mu_{water}$= 9200 cm^{-1}, which approaches the range for an absorption coefficient in protein-rich samples like collagen at the Amide I band [14].

In the experimental data presented in Figure 4 of the main text, an almost 5-fold signal difference between AB and surrounding tissue is observed. For the geometry in Figure S2B, a 5:1 ratio of the absorption coefficients was assumed for the interface, with μ_{-AB} remaining the same as previously at μ_{AB} =6900 cm^{-1} and $\mu_{ST}=0.2^*\ \mu_{AB}$ =1380 cm^{-1} (see Figure S2B where water is replaced by surrounding tissue with densely packed spherical features whose radii were set equal to 400 nm, as shown in orange). The lower signal of the ST features can be attributed to a higher concentration of non-absorbing components like lipid droplets and other membranous features at the wavenumber of 1660 cm^{-1}, resulting in less chromophore presence. Similarly, to the experimental analysis presented in Figure 4 of the main text for point ST, the region in the surrounding tissue distanced 1 μm from the interface is investigated and referred to as point STF.

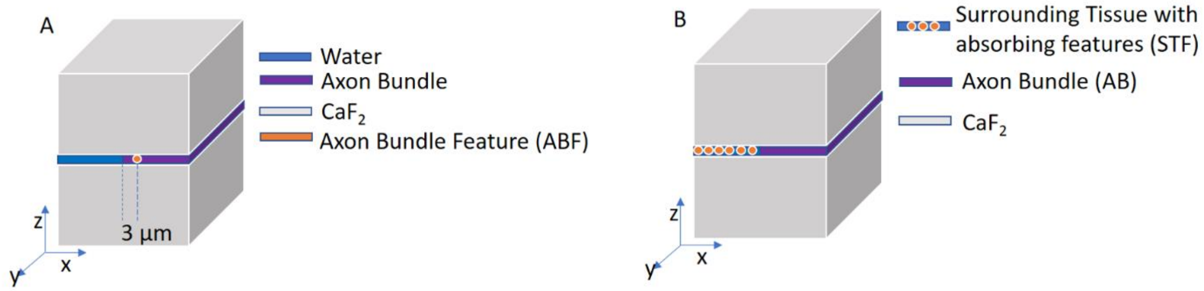

Table 1

Parameters	Units	Water	Axon Bundle	CaF_2	Axon Bundle Feature	Surrounding Tissue Features
Absorption coefficient	μ (cm^{-1})	2300	6900	0	9200	1380
Thermal conductivity	κ ($W{\cdot}m^{-1}{\cdot}K^{-1}$)	0.6	0.6	9.71	0.15	0.15
Volumetric Heat Capacity	C_v ($MJ{\cdot}m^{-3}{\cdot}K^{-1}$)	4.2	4.2	2.71	2.4	2.4

Figure S2. (A). Simulation cuboid geometry (with 200 nm step size) of thin aqueous layer of Water and axon bundle interface sandwiched between two thick CaF_2 windows with ABF point distanced 3 μm from the interface. (B) Simulation cuboid geometry (with 100 nm step size) of thin aqueous layer of surrounding tissue and axon bundle interface sandwiched between two thick CaF_2 windows with STF point distanced 1 μm from the interface. Table 1 includes simulation parameters used for each region in the two cuboids.

In Figure S3A the temperature profile at t=10 ns is presented for a 3:1 absorption ratio (μ-$_{water}$ =2300 cm^{-1} and μ-$_{ABF}$ =6900 cm^{-1}), demonstrating the initial temperature distribution before any significant diffusion has occurred. After t=500 ns, during the diffusion window between t=0.75 μs and t=1.25 μs (see Figure S3B), a strong temperature decay from points ABF_1 and ABF_2 is visible while the signal at point Water (distanced 1 μm from the interface) also decreases. The time curves of points ABF_1, ABF_2 and Water are shown in Figure S3C. It is observed that a faster decay is found for point ABF_1 equal to $\tau_{ABF\text{-}1}$=900 ns, compared to the time decay parameter of point Water of τ_{water} =1.8 μs. A slower decay of $\tau_{ABF\text{-}2}$=2.2 ns is found for feature ABF_2 compared to water. This difference is attributed to the inherent different sizes of the ABF features. It can be thus concluded that even though two regions can be characterized by similar chemical composition and thermal properties, the dimensions play a critical role in the transient thermal dynamics.

The rate of transfer (*RT*) value at time points (t=0.75 μs, t=1 μs and t=1.25 μs) was calculated which, as mentioned in the main text, is defined as the ratio of the time derivative of the temperature over the Laplacian of the spatial temperature profile, for each of the three selected points in time. It can be seen that $RT_{ABF\text{-}1}$ and $RT_{ABF\text{-}2}$ have a mean value at $3.5{\cdot}10^{-7}$ m^2/s and $2.3{\cdot}10^{-7}$ m^2/s, while RT_{water} has an almost 1.5 times larger value at $6.6{\cdot}10^{-7}$ m^2/s (see Figure S3D). This matches the experimental conditions presented in Figure 3 of the main text, where the RT value at the water side of the interface was larger than the hotspots A1 and A2 by almost a factor of 1.8.

Different interface dynamics are demonstrated for the case of the AB – Surrounding Tissue interface (see Figure S3E). Firstly, the larger temperature difference between the two sides compared to the previous scenario results in a higher level of thermal confinement observed at the interface gradient which is sustained throughout the diffusion process. This is characterized by a double exponential decay in the temperature time curves for point STF, presented in Figure S3G (with an initial fast decay for t<1μs and succeeding slower time decay parameter for t>1μs). The initial fast decay can be directly correlated to the inherent absorption of the sphere and its small size, while after t>1μs heat from the neighboring AB starts diffusing towards STF, slowing down significantly its overall decay rate. Overall, for t>1μs, the time decay parameter measured at STF are shown to be larger than at the neighboring AB point, indicating enhanced thermal resistance at the lower temperature side of the interface. As a result, the calculated *RT* value of point STF, with

RT_{STF}=1·10^{-7} m^2/s (for the case of r=400 nm) is 1.6 orders of magnitude times smaller than the *RT* value measured at point AB with RT_{AB}=16·10^{-7} m^2/s (see Figure S3H). This difference in *RT* value is similar to what is seen in the experimental results presented in Figure 4 of the main text. This confirms that the combination of relatively large initial temperature difference as well as the higher curvature of the absorbing features in the surrounding tissue region contribute to enhanced thermal interface resistance.

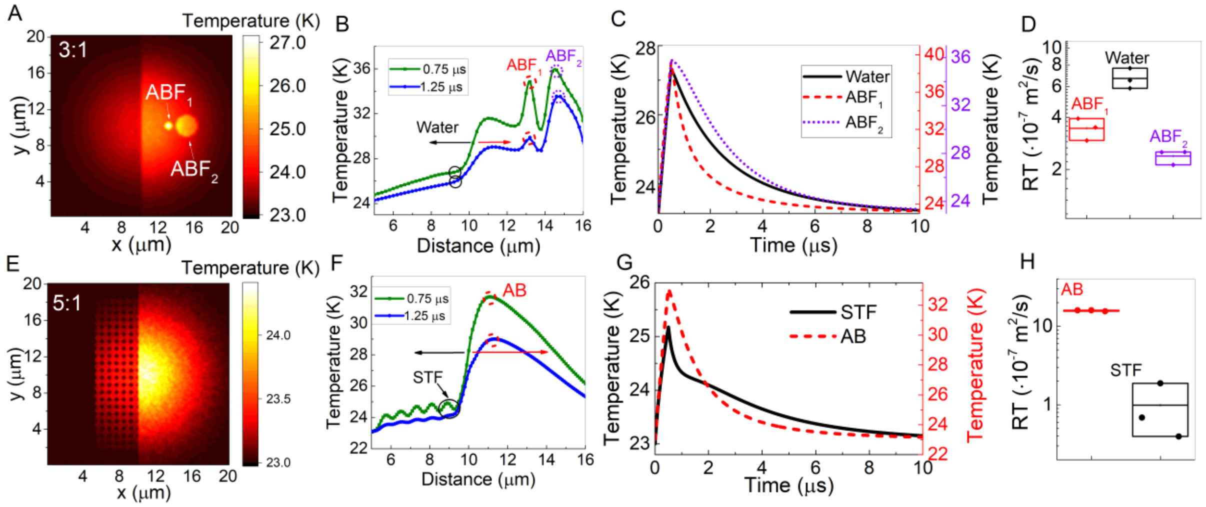

Figure S3. (A) Temperature profile at t= 10 ns of the axon bundle-water interface for a 3:1 ratio of absorption coefficients. (B) Linescan of temperature profile across axon bundle-water interface (at y=10 µm) for t= 0.75 µs (green) and t= 1.25 µs (blue). (C) Temperature time curves at points ABF$_1$ (dashed red), ABF$_2$ (dotted purple) and Water (solid black). (D) Box chart graph of rate of transfer values at points ABF$_1$ (red), ABF$_2$ (purple) and Water (black). (E) Temperature profile at t= 10 ns of the axon bundle-surrounding tissue interface for a 5:1 ratio of absorption coefficients and 0.4 µm radius of the densely packed absorbing spheres in the surrounding tissue region. (F) Linescan of temperature profile across AB-Surrounding tissue interface (at y=10 µm) for t= 0.75 µs (green) and t= 1.25 µs (blue). (G) Temperature time curve of point AB (dashed red) and point STF (solid black). (H) Box chart graph of rate of transfer values at points AB (red) and STF (black).

SIII. References

(1) Marti, D.; Aasbjerg, R. N.; Andersen, P. E.; Hansen, A. K. MCmatlab: An Open-Source, User-Friendly, MATLAB-Integrated 3D Monte Carlo Light Transport Solver with Heat Diffusion and Tissue Damage. In *Optical Interactions with Tissue and Cells XXX*; Beier, H. T., Ibey, B. L., Eds.; SPIE: San Francisco, United States, 2019; p 27. https://doi.org/10.1117/12.2507754.

(2) Vieweger, M.; Goicochea, N.; Koh, E. S.; Dragnea, B. Photothermal Imaging and Measurement of Protein Shell Stoichiometry of Single HIV-1 Gag Virus-like Nanoparticles. *ACS Nano* **2011**, *5* (9), 7324–7333. https://doi.org/10.1021/nn202184x.

(3) Irvine, W. M.; Pollack, J. B. Infrared Optical Properties of Water and Ice Spheres. *Icarus* **1968**, *8* (1), 324–360. https://doi.org/10.1016/0019-1035(68)90083-3.

(4) LoPachin, R. M.; Castiglia, C. M.; Saubermann, A. J. Elemental Composition and Water Content of Myelinated Axons and Glial Cells in Rat Central Nervous System. *Brain Research* **1991**, *549* (2), 253–259. https://doi.org/10.1016/0006-8993(91)90465-8.

(5) Lervik, A.; Bresme, F.; Kjelstrup, S.; Bedeaux, D.; Rubi, J. M. Heat Transfer in Protein–Water Interfaces. *Phys. Chem. Chem. Phys.* **2010**, *12* (7), 1610–1617. https://doi.org/10.1039/B918607G.

(6) Xue, Y.; Lofland, S.; Hu, X. Thermal Conductivity of Protein-Based Materials: A Review. *Polymers (Basel)* **2019**, *11* (3), 456. https://doi.org/10.3390/polym11030456.

(7) Cooper, A. Protein Heat Capacity: An Anomaly That Maybe Never Was. *J. Phys. Chem. Lett.* **2010**, *1* (22), 3298–3304. https://doi.org/10.1021/jz1012142.

(8) Fischer, H.; Polikarpov, I.; Craievich, A. F. Average Protein Density Is a Molecular-Weight-Dependent Function. *Protein Sci* **2004**, *13* (10), 2825–2828. https://doi.org/10.1110/ps.04688204.

(9) Youssefian, S.; Rahbar, N.; Lambert, C. R.; Van Dessel, S. Variation of Thermal Conductivity of DPPC Lipid Bilayer Membranes around the Phase Transition Temperature. *Journal of The Royal Society Interface* **2017**, *14* (130), 20170127. https://doi.org/10.1098/rsif.2017.0127.

(10) Nakano, T. (中野雄大); Kikugawa, G. (菊川豪太); Ohara, T. (小原拓). A Molecular Dynamics Study on Heat Conduction Characteristics in DPPC Lipid Bilayer. *The Journal of Chemical Physics* **2010**, *133* (15), 154705. https://doi.org/10.1063/1.3481650.

(11) Gullapalli, R. R.; Demirel, M. C.; Butler, P. J. Molecular Dynamics Simulations of DiI-C18(3) in a DPPC Lipid Bilayer. *Phys. Chem. Chem. Phys.* **2008**, *10* (24), 3548–3560. https://doi.org/10.1039/B716979E.

(12) Blume, A. Apparent Molar Heat Capacities of Phospholipids in Aqueous Dispersion. Effects of Chain Length and Head Group Structure. *Biochemistry* **1983**, *22* (23), 5436–5442. https://doi.org/10.1021/bi00292a027.

(13) Bastos, A. R. N.; Brites, C. D. S.; Rojas-Gutierrez, P. A.; DeWolf, C.; Ferreira, R. A. S.; Capobianco, J. A.; Carlos, L. D. Thermal Properties of Lipid Bilayers Determined Using Upconversion Nanothermometry. *Advanced Functional Materials* **2019**, *29* (48), 1905474. https://doi.org/10.1002/adfm.201905474.

(14) Scholkmann, F.; Kleiser, S.; Metz, A. J.; Zimmermann, R.; Mata Pavia, J.; Wolf, U.; Wolf, M. A Review on Continuous Wave Functional Near-Infrared Spectroscopy and Imaging Instrumentation and Methodology. *NeuroImage* **2014**, *85*, 6–27. https://doi.org/10.1016/j.neuroimage.2013.05.004.
